# Supplementary material for: Polyketide synthase gene clusters in the ericoid mycorrhizal fungus Oidiodendron maius: genome-wide prediction, expression profiling, and functional characterization of knockout mutants
Source: Front Plant Sci. 2026 Sep 16;17:1924430. doi: 10.3389/fpls.2026.1924430 (PMC13623903; doi:10.3389/fpls.2026.1924430)
Supplement: Supplementary file 1 [file SupplementaryFile1.zip › Supplementary Materials/Supplementary Folder 1.zip!/Oidma1_AssemblyScaffolds_Repeatmasked/knownclusterblast/cluster43/jgi.p_Oidma1_183917_mibig_hits.html]

| MIBiG Protein | Description | MIBiG Cluster | MiBiG Product | % ID | % Coverage | BLAST Score | E-value |
| --- | --- | --- | --- | --- | --- | --- | --- |
| CRG85577.1 | hypothetical\_protein | BGC0001402 | Cyclochlorotine\_biosynthetic\_gene\_cluster | 29 | 96.2938451357 | 542 | 1.2e-153 |
| AMM63163.1 | AniL | BGC0001371 | Echinocandin\_B\_biosynthetic\_gene\_cluster | 30 | 58.8352084712 | 366 | 1.2e-100 |
| EPE34342.1 | P-loop\_containing\_nucleoside\_triphosphate\_hydrolase | BGC0001035 | Pneumocandin\_biosynthetic\_gene\_cluster | 30 | 59.4970218398 | 363 | 1.3e-99 |
| AFT91383.1 | EcdL | BGC0000338 | Echinocandin\_B\_biosynthetic\_gene\_cluster | 30 | 60.1588352085 | 359 | 1.9e-98 |
| OAQ83754.1 | ABC\_multidrug\_transporter | BGC0001358 | Leucinostatins\_biosynthetic\_gene\_cluster | 27 | 62.342819325 | 288 | 5.3e-77 |
| AAN74822.1 | Fum19p | BGC0000062 | Fumonisin\_biosynthetic\_gene\_cluster | 26 | 67.9682329583 | 277 | 1.2e-73 |
| ACB12564.1 | Fum19 | BGC0000063 | Fumonisin\_biosynthetic\_gene\_cluster | 26 | 63.5340833885 | 251 | 7.2e-66 |
| ADX66465.1 | ScnB | BGC0000108 | Natamycin\_biosynthetic\_gene\_cluster | 29 | 32.9583057578 | 161 | 9.8e-39 |
| CAC20925.1 | PimB\_protein | BGC0000125 | Pimaricin\_biosynthetic\_gene\_cluster | 30 | 33.1568497684 | 160 | 1.3e-38 |
| ACQ63630.1 | ABC\_multidrug\_resistance\_transporter | BGC0000875 | Caprazamycin\_biosynthetic\_gene\_cluster | 29 | 35.0761085374 | 153 | 2.7e-36 |
| CAG38700.1 | putative\_ABC-transporter,\_ATP-binding\_protein | BGC0000708 | Lividomycin\_biosynthetic\_gene\_cluster | 26 | 35.6055592323 | 150 | 1.3e-35 |
| CAF31837.1 | putative\_hygromycin\_B\_exporter | BGC0000699 | Hygromycin\_B\_biosynthetic\_gene\_cluster | 26 | 35.8041032429 | 148 | 6.6e-35 |
| AAF71763.1 | NysG | BGC0000115 | Nystatin\_biosynthetic\_gene\_cluster | 27 | 31.8332230311 | 147 | 1.1e-34 |
| CAA12529.1 | ExsA\_protein | BGC0000802 | Succinoglycan\_biosynthetic\_gene\_cluster | 29 | 26.075446724 | 145 | 5.6e-34 |
| BAJ05895.1 | putative\_ABC\_multidrug\_resistance\_transporter | BGC0000872 | A-90289\_biosynthetic\_gene\_cluster | 29 | 34.9437458637 | 143 | 2.1e-33 |
| CAI47638.1 | putative\_ABC-type\_aminoglycoside\_exporter | BGC0000692 | Apramycin\_biosynthetic\_gene\_cluster | 25 | 39.8411647915 | 141 | 6.1e-33 |
| CAF32376.1 | putative\_ABC\_transporter,\_ATP-binding\_protein | BGC0000712 | Paromomycin\_biosynthetic\_gene\_cluster | 26 | 38.7822634017 | 141 | 1e-32 |
| CAC20924.1 | PimA\_protein | BGC0000125 | Pimaricin\_biosynthetic\_gene\_cluster | 30 | 25.7445400397 | 139 | 3e-32 |
| CAD89782.1 | hypothetical\_protein | BGC0001010 | Melithiazol\_biosynthetic\_gene\_cluster | 27 | 33.2892124421 | 136 | 2.6e-31 |
| ADX66464.1 | ScnA | BGC0000108 | Natamycin\_biosynthetic\_gene\_cluster | 30 | 25.7445400397 | 136 | 3.4e-31 |
| ABV83218.1 | CppG | BGC0000116 | Nystatin-like\_Pseudonocardia\_polyene\_biosynthetic\_gene\_cluster | 27 | 33.4215751158 | 135 | 5.7e-31 |
| AAK73498.1 | AmphG | BGC0000015 | Amphotericin\_biosynthetic\_gene\_cluster | 26 | 32.5612177366 | 134 | 1.3e-30 |
| CAJ88629.1 | putative\_netropsin\_resistance\_protein\_subunit\_2 | BGC0000327 | Congocidine\_biosynthetic\_gene\_cluster | 28 | 33.4877564527 | 133 | 2.2e-30 |
| CAB38879.1 | putative\_ABC\_transporter | BGC0000315 | Calcium-dependent\_antibiotic\_biosynthetic\_gene\_cluster | 27 | 35.5393778954 | 133 | 2.9e-30 |
| CAI47637.1 | putative\_ABC-type\_aminoglycoside\_exporter | BGC0000692 | Apramycin\_biosynthetic\_gene\_cluster | 26 | 35.737921906 | 133 | 2.9e-30 |
| CAG38699.1 | putative\_ABC-transporter,\_ATP-binding\_protein | BGC0000708 | Lividomycin\_biosynthetic\_gene\_cluster | 26 | 32.7597617472 | 132 | 3.7e-30 |
| BAI23327.1 | putative\_ABC-transporter | BGC0000949 | A-500359s\_biosynthetic\_gene\_cluster | 27 | 34.6790205162 | 132 | 3.7e-30 |
| ADC96668.1 | ABC\_transporter | BGC0001076 | Liposidomycin\_biosynthetic\_gene\_cluster | 28 | 35.8702845797 | 131 | 6.4e-30 |
| AGL93176.1 | ABC\_transporter\_ATP-binding\_protein | BGC0000560 | Subtilomycin\_biosynthetic\_gene\_cluster | 25 | 39.5764394441 | 131 | 8.3e-30 |
| AHH99931.1 | ABC\_transporter\_protein | BGC0000002 | Aculeximycin\_biosynthetic\_gene\_cluster | 30 | 20.9133024487 | 129 | 2.4e-29 |
| CAF31838.1 | putative\_hygromycin\_B\_exporter | BGC0000699 | Hygromycin\_B\_biosynthetic\_gene\_cluster | 26 | 33.6863004633 | 128 | 5.4e-29 |
| ACA97579.1 | PmxD | BGC0000408 | Polymyxin\_biosynthetic\_gene\_cluster | 24 | 33.7524818001 | 128 | 9.2e-29 |
| AAX31531.1 | putative\_ABC\_transporter | BGC0000336 | Daptomycin\_biosynthetic\_gene\_cluster | 24 | 33.1568497684 | 127 | 1.2e-28 |
| BAH43768.1 | ABC\_transporter\_ATP-binding/permease\_protein | BGC0000452 | Tyrocidine\_biosynthetic\_gene\_cluster | 25 | 32.8259430841 | 127 | 1.6e-28 |
| ABV83219.1 | CppH | BGC0000116 | Nystatin-like\_Pseudonocardia\_polyene\_biosynthetic\_gene\_cluster | 30 | 25.8769027134 | 127 | 1.6e-28 |
| BAB04170.1 | lantibiotic\_mersacidin\_transporter\_system | BGC0000517 | Haloduracin\_alpha\_/\_haloduracin\_beta\_biosynthetic\_gene\_cluster | 25 | 34.7452018531 | 127 | 1.6e-28 |
| AEZ51519.1 | PmxD | BGC0001153 | Polymyxin\_biosynthetic\_gene\_cluster | 24 | 33.7524818001 | 126 | 2e-28 |
| AMR44284.1 | ABC\_transporter | BGC0001398 | Phomopsins\_biosynthetic\_gene\_cluster | 22 | 63.8649900728 | 126 | 2.7e-28 |
| BAE07080.1 | possible\_ABC\_transporter | BGC0000693 | Butirosin\_biosynthetic\_gene\_cluster | 24 | 36.2011912641 | 126 | 3.5e-28 |
| AJM89737.1 | PmxD | BGC0001192 | Colistin\_biosynthetic\_gene\_cluster | 24 | 33.7524818001 | 125 | 5.9e-28 |
| AET14838.1 | multidrug\_resistance\_protein | BGC0001274\_c2 | Non-acetylated\_open-chain\_sophorolipid\_/\_diacetylated\_lactonic | 31 | 21.4427531436 | 124 | 1e-27 |
| ALV82355.1 | ABC\_transporter | BGC0001370 | Lipopeptide\_8D1-1\_/\_lipopeptide\_8D1-2\_biosynthetic\_gene\_cluster | 28 | 35.6055592323 | 123 | 1.7e-27 |
| AGM16416.1 | paenibacterin\_ABC-transporter\_E | BGC0000400 | Paenibacterin\_biosynthetic\_gene\_cluster | 24 | 35.0099272005 | 123 | 1.7e-27 |
| BAL72550.1 | lariatin\_biosynthetic\_protein | BGC0000575 | Lariatin\_biosynthetic\_gene\_cluster | 30 | 20.1853077432 | 123 | 2.3e-27 |
| ANP43732.1 | ThsT | BGC0001391 | Thusin\_biosynthetic\_gene\_cluster | 26 | 35.8041032429 | 122 | 3.9e-27 |
| WP\_006126653.1 | ABC\_transporter | BGC0000554 | SRO15-3108\_biosynthetic\_gene\_cluster | 28 | 31.7670416942 | 122 | 5e-27 |
| CCC55903.1 | putative\_ABC\_transporter,\_ATPase\_and\_permease\_component | BGC0000973 | Collismycin\_A\_biosynthetic\_gene\_cluster | 30 | 22.1707478491 | 121 | 1.1e-26 |
| XP\_011392729.1 | ABC\_transporter | BGC0001281 | Ustilagic\_acid\_biosynthetic\_gene\_cluster | 28 | 24.0238252813 | 120 | 1.9e-26 |
| CAC17507.1 | putative\_ABC\_transporter\_ATP-binding\_protein | BGC0000324 | Coelibactin\_biosynthetic\_gene\_cluster | 26 | 34.0833884844 | 119 | 4.3e-26 |
| AFD30962.1 | CrmT3 | BGC0000966 | Caerulomycin\_A\_biosynthetic\_gene\_cluster | 29 | 31.5684976837 | 118 | 7.3e-26 |
| AGM16415.1 | paenibacterin\_ABC-transporter\_D | BGC0000400 | Paenibacterin\_biosynthetic\_gene\_cluster | 23 | 36.3335539378 | 117 | 1.6e-25 |
| EFI65097.1 | lantibiotic\_transport\_protein | BGC0001407 | Bicereucin\_biosynthetic\_gene\_cluster | 26 | 36.5982792852 | 116 | 2.1e-25 |
| CAP20371.1 | ABC\_transporter\_ATP-binding\_membrane\_protein | BGC0000347 | Equibactin\_biosynthetic\_gene\_cluster | 23 | 33.7524818001 | 116 | 2.1e-25 |
| AFJ14797.1 | PlpH | BGC0000403 | Pelgipeptin\_biosynthetic\_gene\_cluster | 25 | 33.5539377895 | 115 | 6.2e-25 |
| ABS50189.1 | putative\_ABC\_transporter\_permease/ATP-binding\_protein | BGC0000410 | Pseudomonine\_biosynthetic\_gene\_cluster | 24 | 32.6273990735 | 115 | 6.2e-25 |
| ALK27913.1 | putative\_ABC\_transporter | BGC0001233 | Feglymycin\_biosynthetic\_gene\_cluster | 27 | 35.4070152217 | 113 | 2.3e-24 |
| AFY23032.1 | TamA | BGC0000274 | Tetarimycin\_biosynthetic\_gene\_cluster | 27 | 23.0311052283 | 113 | 3.1e-24 |
| EYT83450.1 | ABC\_transporter | BGC0001213 | Nataxazole\_biosynthetic\_gene\_cluster | 29 | 21.7736598279 | 112 | 4e-24 |
| BAH43767.1 | ABC\_transporter\_ATP\_binding/permease\_protein | BGC0000452 | Tyrocidine\_biosynthetic\_gene\_cluster | 24 | 39.7088021178 | 110 | 1.5e-23 |
| AAG28766.1 | CoaD | BGC0000617 | Coagulin\_biosynthetic\_gene\_cluster | 24 | 36.5320979484 | 110 | 1.5e-23 |
| AFN69431.1 | ElxT | BGC0000509 | Epilancin\_15X\_biosynthetic\_gene\_cluster | 24 | 32.2303110523 | 106 | 2.2e-22 |
| CAD15510.1 | probable\_composite\_atp-binding\_transmembrane\_abc\_transporter\_protein | BGC0001014 | Micacocidin\_biosynthetic\_gene\_cluster | 25 | 32.8259430841 | 102 | 3.2e-21 |
| AHW81482.1 | hypothetical\_protein | BGC0000934 | Endophenazines\_biosynthetic\_gene\_cluster | 28 | 22.236929186 | 101 | 7e-21 |
| CAP20370.1 | ABC\_transporter,\_ATP-binding\_membrane\_protein | BGC0000347 | Equibactin\_biosynthetic\_gene\_cluster | 25 | 30.1125082727 | 100 | 1.6e-20 |
| AAG32538.1 | SalT | BGC0000548 | Salivaricin\_A\_biosynthetic\_gene\_cluster | 21 | 32.8921244209 | 100 | 1.6e-20 |
| ABQ84449.1 | salivaricin\_ABC-transporter | BGC0000624 | Salivaricin\_CRL1328\_alpha\_peptide\_/\_salivaricin\_CRL1328\_beta\_peptide | 24 | 33.4215751158 | 100 | 2e-20 |
| AFD30963.1 | CrmT2 | BGC0000966 | Caerulomycin\_A\_biosynthetic\_gene\_cluster | 25 | 29.3845135672 | 99 | 2.7e-20 |
| AAC63532.1 | sublancin\_transporter\_protein | BGC0000558 | Sublancin\_168\_biosynthetic\_gene\_cluster | 23 | 35.8702845797 | 97 | 1e-19 |
| AAA62651.1 |  | BGC0000504 | Cytolysin\_ClyLl\_/\_cytolysin\_ClyLs\_biosynthetic\_gene\_cluster | 24 | 36.3335539378 | 97 | 1.7e-19 |
| CAA90021.1 | PepT | BGC0000543 | Pep5\_biosynthetic\_gene\_cluster | 23 | 32.4288550629 | 96 | 2.3e-19 |
| AEH59056.1 | YvcC/ABC\_transporter | BGC0000385 | Lysobactin\_biosynthetic\_gene\_cluster | 23 | 35.0099272005 | 96 | 3.9e-19 |
| CAX48974.1 | ABC-transporter | BGC0000519 | Labyrinthopeptin\_A1,A3\_/\_labyrinthopeptin\_A2\_biosynthetic\_gene | 28 | 26.075446724 | 95 | 6.6e-19 |
| AHX39584.1 | ABC\_transporter\_protein | BGC0000626 | Thuricin\_biosynthetic\_gene\_cluster | 22 | 33.8848444739 | 94 | 8.6e-19 |
| ACP43452.1 | McnB | BGC0000590 | Microcin\_N\_biosynthetic\_gene\_cluster | 23 | 31.2375909993 | 94 | 1.5e-18 |
| BAU50927.1 | ABC\_transporter | BGC0001379 | BD-12\_biosynthetic\_gene\_cluster | 24 | 33.0906684315 | 90 | 2.1e-17 |
| BAG19224.1 | membrane\_translocator | BGC0000496 | AmfS\_biosynthetic\_gene\_cluster | 23 | 34.9437458637 | 87 | 1.8e-16 |
| CAC17506.1 | putative\_ABC\_transporter\_ATP-binding\_protein | BGC0000324 | Coelibactin\_biosynthetic\_gene\_cluster | 25 | 32.6935804103 | 82 | 5.8e-15 |
| AAD56148.1 | MutT | BGC0000533 | Mutacin\_III\_biosynthetic\_gene\_cluster | 24 | 28.3256121774 | 81 | 9.8e-15 |
| EYT83451.1 | ABC\_transporter\_ATP-binding\_protein | BGC0001213 | Nataxazole\_biosynthetic\_gene\_cluster | 23 | 33.9510258107 | 80 | 2.2e-14 |
| CAC60277.1 | RumT\_protein | BGC0000545 | Ruminococcin\_A\_biosynthetic\_gene\_cluster | 21 | 29.5830575778 | 67 | 1.1e-10 |
| ACY56719.1 | ThnT | BGC0000600 | Thurincin\_H\_biosynthetic\_gene\_cluster | 24 | 20.3176704169 | 58 | 6.8e-08 |
| CAM03501.1 | putative\_ABC\_transporter | BGC0000513 | Erythreapeptin\_biosynthetic\_gene\_cluster | 22 | 30.3772336201 | 53 | 2.9e-06 |
